# Differences Between the Unipolar Versus Bipolar Potential‐Based Activation Maps of Ventricular Premature Contractions Arising From Ventricular Outflow Tracts

**DOI:** 10.1111/jce.16647

**Published:** 2025-03-23

**Authors:** Yoshimori J. An, Masafumi Sugawara, Jakub Sroubek, Katsuhide Hayashi, John O. Lopez, Justin Lee, Shady Nakhla, Pasquale Santangeli, Oussama M. Wazni, Koji Higuchi

**Affiliations:** ^1^ Cardiac Electrophysiology Section, Department of Cardiovascular Medicine Cleveland Clinic Cleveland Ohio USA

**Keywords:** 3‐dimensional mapping, automatic annotation, bipolar electrogram, local activation time, unipolar electrogram, ventricular premature contraction

## Abstract

**Background:**

The use of an automated annotation algorithm based on the maximal negative derivative of the unipolar potential (−dV/dTmax) for local activation timing in the ablation of outflow tract (OT) ventricular premature contractions (VPCs) remains controversial.

**Objective:**

To investigate the spatial differences in the earliest activation sites (EASs) of OT‐VPCs identified by an automated annotation based on unipolar −dV/dTmax versus manual annotation using local bipolar potentials.

**Methods:**

Seventy‐nine patients with frequent OT‐VPCs who underwent successful ablation were included. VPCs originated from the right ventricular OT (RVOT) free wall (*n* = 10), RVOT septum (*n* = 25), aortomitral continuity (AMC) (*n* = 19), and aortic sinus cusps (ASCs) (*n* = 25). The spatial distance between EASs identified by the two annotation methods was analyzed.

**Results:**

The spatial distance between EASs was significantly larger in ASC‐origin VPCs compared to non‐ASC‐origin VPCs (median: 11.9 mm [IQR: 7.9–14.9] vs. 1.2 mm [IQR: 0.0–3.3], *p* < 0.001). Among non‐ASC‐origin VPCs, the spatial difference was smallest in VPCs from the RVOT free wall (median: 0 mm) and larger in those from the RVOT septum (median: 1.6 mm) and AMC (median: 2.2 mm).

**Conclusion:**

The spatial discordance of EAS between unipolar and bipolar mapping varies by the VPC origin site. The discrepancy is particularly pronounced in ASC‐origin VPCs, emphasizing the need for careful interpretation of automated annotation algorithms to ensure accurate localization and effective ablation.

## 1 Introduction

An automatic local activation time (LAT) annotation algorithm is a key component of contemporary 3D electro‐anatomical mapping systems during catheter ablation procedures. Typically, the timing of local activation is assessed to the point of a maximal negative derivative of the unipolar potential (−dV/dTmax), a concept rooted in the idea that the maximal intracellular voltage gradient at the leading edge of a propagating wavefront corresponds to an inverse change in the extracellular potential [1]. This classical concept was largely derived from data obtained from 2D muscle preparations [2]. How well this concept extrapolates into the 3D space is less clear, particularly when using unipolar −dV/dTmax as a means of identifying accurate ablation sites for ventricular premature contractions (VPCs). Recent studies have shown that the first deflection of the local bipolar electrogram may be more useful than unipolar −dV/dTmax in this clinical context [3, 4]. However, there is still limited data on how different the 3D activation mappings of the targeted VPCs are in comparison between those by these two different annotation algorithms for the local activation timing and how different they are based on the location of VPCs.

The present study aims to investigate the differences in the earliest activation sites (EASs) of VPC originating from the right and left ventricular outflow tracts (OTs), using an automated annotation algorithm based on unipolar −dV/dTmax versus a manual annotation of local bipolar potential.

## 2 Methods

### 2.1 Study Design and Patient Population

This retrospective study included patients who underwent a successful ablation of VPC arising from the right or left ventricular OT without recurrence at discharge between May 2018 and April 2022 at the Cleveland Clinic. Only patients with high‐quality electrograms and frequent baseline VPCs (in bigeminy, trigeminy, or quadrigeminy patterns) were included to ensure accurate analysis of the response to RF energy delivery. To minimize confounders, we limited our study to procedures done using a single mapping system (CARTO 3 system [Biosense Webster, Diamond Bar, California]). A successful ablation site was defined as a location where ablation resulted in complete and durable VPC elimination, with no recurrence for ≥ 30 min following ablation, despite using provocative measures such as programmed electrical stimulation and administration of isoproterenol, calcium chloride, or atropine. The origin of the VPC was classified based on the successful ablation site and categorized as either right‐ or left‐sided depending on where elimination was achieved when ablation was performed from both sides. Cases in which VPCs were not eliminated or required epicardial ablation (including from coronary veins) were excluded. Additionally, VPCs associated with structural heart diseases, such as prior myocardial infarction or surgical scars, were also excluded. All patients provided informed consent for the procedure, and the Cleveland Clinic Foundation Institutional Review Board independently approved the data analysis. This study adheres to the principles outlined in the Declaration of Helsinki (7th revision).

### 2.2 Electrophysiological Study and Ablation

All patients underwent catheter ablation using the CARTO3 system (Biosense Webster, Diamond Bar, California) for guidance. Mapping catheters included either a system‐compatible 3.5‐mm mapping/ablation catheter or a multielectrode mapping catheter. In the standard automated algorithm incorporated into the CARTO system, LAT is determined using a combination of bipolar and unipolar electrograms, with time annotation made at the unipolar −dV/dTmax that coincides with a bipolar potential [5]. The reference to perform the mapping based on LAT was set as an ECG surface lead with a well‐defined R‐wave peak (R peak = 0) or the signal's center of energy of the body surface ECG precordial channels (V1–V6) using the so‐called Advanced Reference Annotation algorithm at the operator's discretion. The nominal high‐ and low‐pass filter corner frequencies were set to 16 and 500 Hz for bipolar electrograms and 2 and 240 Hz for unipolar electrograms, respectively. It is our practice to turn off the notch filter. Intracardiac echocardiography was used in all cases.

For each case, the sequence of ablation applications was retrieved from the CARTO system log, and the location of the successful application, including its order in the total list of applications, was identified. Cases were excluded from analysis if these data were lacking. In all cases, ablation was performed at the EAS based on the local bipolar potential with an irrigated catheter with a power output of 30–45 W and a maximum temperature limit of 43°C.

### 2.3 Electrogram Analysis

**Figure 1:**
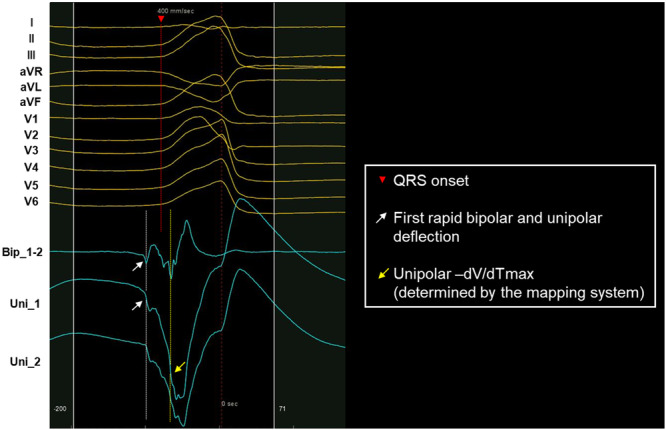
This illustrates local electrogram analysis. A 12‐lead electrocardiogram of a ventricular premature contraction (VPC) with bipolar and unipolar electrograms is shown at a sweep speed of 400 mm/s. The white dotted line indicates the timing of the first rapid bipolar deflection, which was annotated manually. This corresponds to the first rapid unipolar deflection. The red dotted line indicates the QRS onset, and the yellow dotted line indicates the timing of the unipolar −dV/dTmax as determined by the automated algorithm in the mapping system. −dV/dTmax = maximal negative derivative of the extracellular unipolar potential; VPC = ventricular premature contraction.

Endocardial electrograms at the sites of VPC suppression in successful cases were reviewed and analyzed using the CARTO system at a sweep speed of 400 mm/s. At each site, both bipolar and unipolar electrograms were evaluated, and the following parameters and intervals were measured: (1) the interval from the first rapid bipolar deflection to QRS onset, which was defined as the LAT based on the bipolar potential, (2) the interval from the unipolar −dV/dTmax to QRS onset (where −dV/dTmax was determined by the mapping system), and (3) the interval from the first rapid bipolar deflection to the unipolar −dV/dTmax. To ensure the accuracy of bipolar annotation, the unipolar signal was carefully examined alongside the bipolar signal to confirm the true first rapid bipolar deflection. Specifically, when manually annotating small bipolar potentials that were not automatically annotated but likely represent local activation, we closely assessed the corresponding unipolar signal. If no corresponding unipolar signal was observed or if the bipolar signal was not reproducible, it was considered noise. Figure 1 illustrates an example of the discordance between manual bipolar annotation and automated annotation, demonstrating how these were annotated. To assess inter‐observer reliability, the first rapid bipolar deflection at 40 randomly selected points was re‐annotated by a second independent observer. Intra‐observer agreement was also evaluated by re‐annotating these points by the same observer. All observers were blinded to the previous measurements to ensure unbiased assessment.

For each patient, two activation maps of the targeted VPC were created using two methods: automated annotation based on unipolar −dV/dTmax and manual annotation based on the first rapid bipolar deflection. The EAS was identified in both unipolar‐based and bipolar‐based maps, and the distance between the two EASs was measured using the CARTO system's distance measurement tool. The study population was then divided into two groups based on VPC origin: those with aortic sinus cusp (ASC) origin and those with non‐ASC origin, including the right ventricular OT (RVOT) free wall, RVOT septum, and aortomitral continuity (AMC). The VPC origin site was defined as the endocardial bipolar EAS, which corresponded to the site of successful radiofrequency ablation. Finally, we analyzed the differences in the distance between the two EASs, as well as the timing relationship between the first bipolar deflection and unipolar −dV/dTmax, based on the site of VPC origin.

### 2.4 Statistical Analysis

Data were tested for normal (Gaussian) distribution using the one‐sample Kolmogorov–Smirnov test. Continuous variables with a normal distribution were presented as mean ± standard deviation (SD), while non‐Gaussian variables were reported as median with interquartile range (IQR). Categorical variables were expressed as counts and percentages. The Mann–Whitney *U* test was used to compare continuous variables between subgroups based on the ablation outcome and VPC origin. Inter‐observer and intra‐observer agreements for manual annotation of bipolar potentials were assessed by calculating intraclass correlation coefficients (ICCs), with excellent agreement defined as ICC > 0.8. A *p* value of < 0.05 was considered statistically significant. All statistical analyses were performed using R software (version 3.6.2, The R Foundation).

## 3 Results

### 3.1 Patient Characteristics

A total of 569 patients underwent VPC ablation at our institution between May 2018 and April 2022, of which 476 cases utilized the CARTO 3 mapping system. The following inclusion criteria were applied for enrollment: (1) patients with OT‐VPCs that were endocardially ablated; (2) frequent VPCs in bigeminy, trigeminy, or quadrigeminy patterns to allow proper assessment of ablation response; (3) sufficient mapping point density; and (4) adequate electrogram quality for reliable analysis. These criteria narrowed the study population to 79 patients with successfully ablated and well‐mapped OT‐VPCs. The baseline characteristics of these patients are shown in Table 1. The mean VPC burden, measured by a 24‐h Holter monitor before the procedure, was 26.2 ± 10.1%. VPCs originated from the RVOT in 35 patients (44%)—with 10 from the RVOT free wall and 25 from the RVOT septum—and from the left ventricular OT (LVOT) in 44 patients (56%)—with 25 from the ASC and 19 from the AMC. In all cases, a contact force‐sensing ablation catheter was used, with a mean contact force of 13 g (IQR: 9.5–17.0 g) during radiofrequency ablation. Seven cases (9%) needed ablation from both RVOT and LVOT. As long‐term follow‐up data, the mean VPC burden examined around 1 year after the procedure was decreased to 1.0% [IQR: 0.5–1.9].

**Table 1:** Patient characteristics (*n* = 79).

Variable	Value
Age, years	58 ± 15
Male gender	39 (49%)
Hypertension	37 (46%)
Diabetes mellitus	13 (16%)
Coronary artery disease	13 (16%)
LVEF, %	51 (42, 60)
VPC burden (pre‐ablation), %	26.2 ± 10.1
VPC burden (post‐ablation), %	1.0 (0.5, 1.9)
Site of origin, *n* (%)	
RVOT free wall	10 (12%)
RVOT septum	25 (32%)
Aortomitral continuity	19 (24%)
Aortic sinus cusp	25 (32%)
During the ablation procedure	
Ablation power setting (W)	40 (35, 45)
Time to VPC suppression at successful ablation (s)	10.7 ± 8.4
Ablated from both RVOT and LVOT, *n* (%)	7 (9%)

*Notes:* Categorical data are presented as numbers (%). Continuous data are presented as mean ± standard deviation (SD), or median and interquartile range (25%, 75%) according to the distribution.

Abbreviations: LVEF = left ventricular ejection fraction, LVOT = left ventricular outflow tract, RVOT = right ventricular outflow tract, VPC = ventricular premature contraction.

### 3.2 VPCs Originating From Non‐ASC

**Figure 2:**
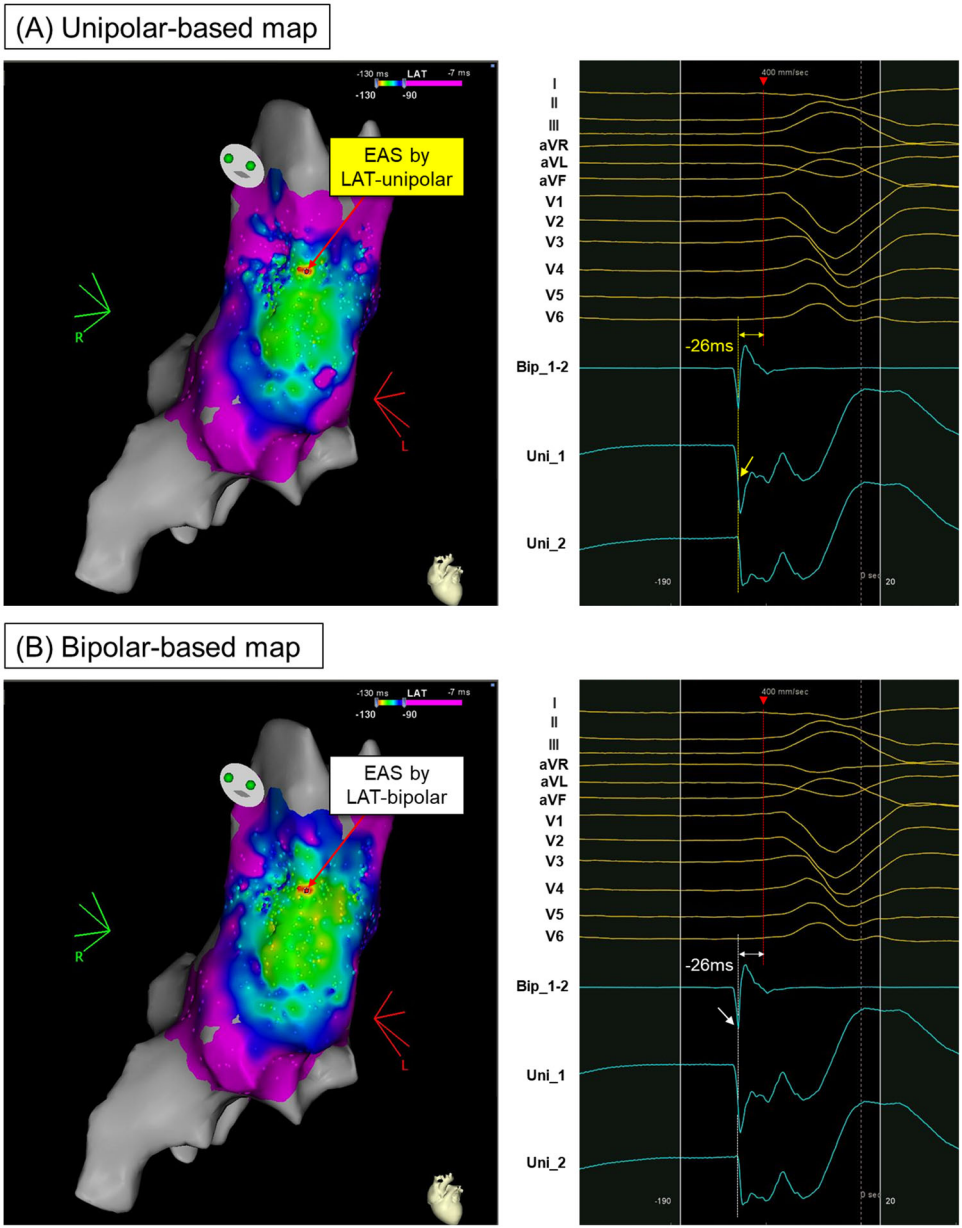
An example of the VPC case arising from RVOT free wall where ablation resulted in immediate suppression. (A) The red arrow in the left panel indicates the earliest activation site (EAS) in the unipolar‐based activation map. The right panel shows the intracardiac signals at this EAS. The yellow dotted line in the right panel indicates the timing with −dV/dTmax determined based on the automated annotation algorithm in the mapping system. It precedes the QRS onset by 26 ms. (B) The red arrow in the left panel indicates the EAS in the bipolar‐based activation map. The right panel shows the intracardiac signals at this EAS. The white dotted line in the right panel indicates the timing of the first bipolar deflection corresponding to the first unipolar deflection. It also precedes the QRS onset by 26 ms. The EASs in both unipolar‐ and bipolar‐based activation maps are identical, which results in the distance between the two EAS of “0 mm.” EAS = earliest activation site; RVOT = right ventricular outflow tract; other abbreviations as in Figures 1 and 2.

**Figure 3:**
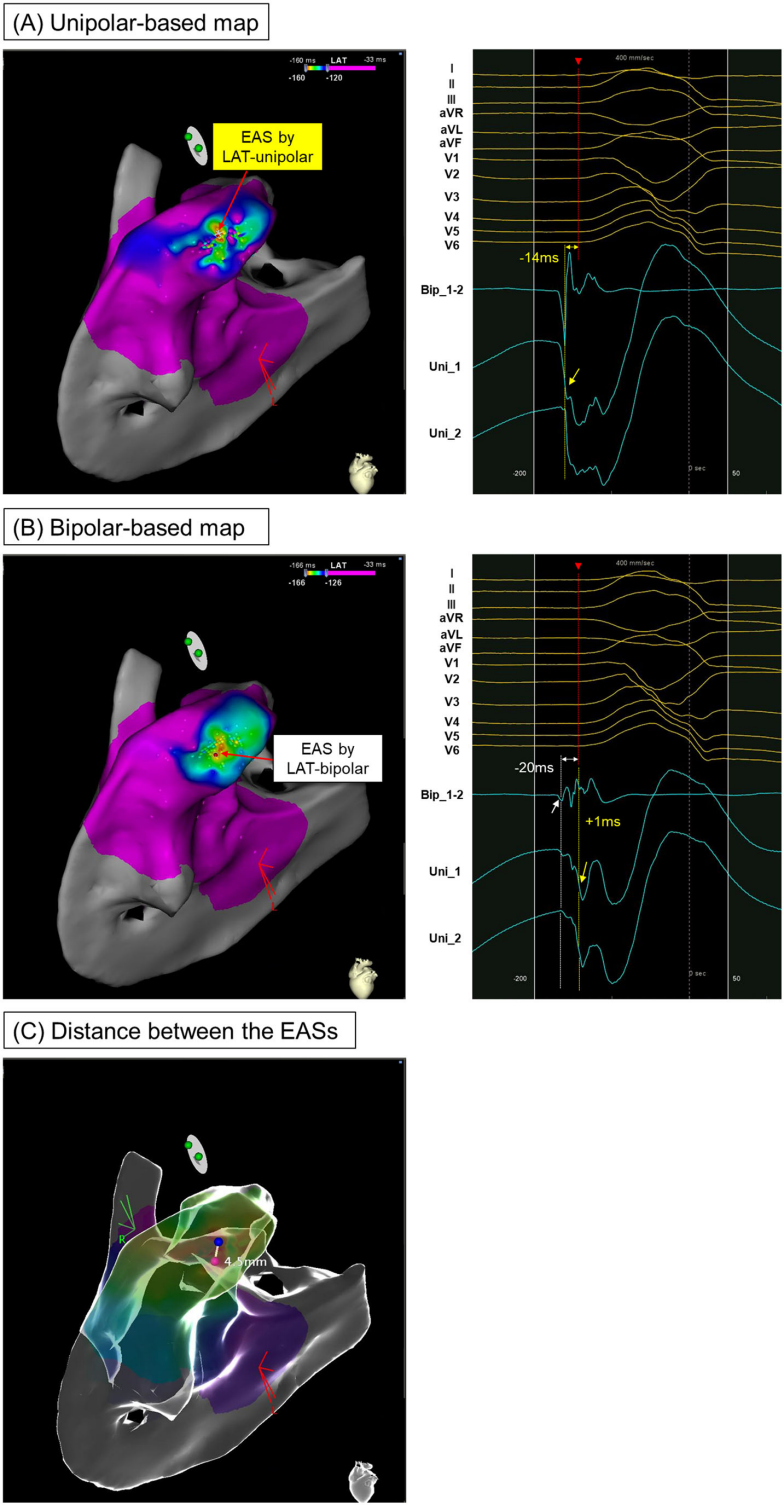
An example of the VPC case arising from the septal RVOT where ablation resulted in delayed suppression. (A) The red arrow in the left panel indicates the EAS in the unipolar‐based activation map. The right panel shows the intracardiac signals at this EAS. The timing with the unipolar −dV/dTmax (yellow dotted line) preceded the QRS onset by 14 ms. (B) The red arrow in the left panel indicates the EAS in the bipolar‐based activation map. The right panel shows the intracardiac signals at this EAS. The first bipolar deflection (white dotted line) precedes the QRS onset by 26 ms, corresponding to the first unipolar deflection. The unipolar −dV/dTmax occurs 23 ms later than this first bipolar deflection (i.e., 3 ms ahead of the QRS onset). (C) The blue and pink tags denote the EASs in the unipolar‐ and bipolar‐based activation maps, respectively. The distance between these points was 4.5 mm. Abbreviations as in Figures 1, 2, 3.

Figure 2 illustrates a VPC originating from the RVOT free wall, highlighting the timing and spatial relationship between the bipolar and unipolar potential‐based mappings. In this case, the EASs in both the unipolar and bipolar maps were identical, resulting in a distance of “0 mm” between the two EASs. In contrast, Figure 3 depicts a VPC originating from the RVOT septum. Here, the unipolar −dV/dTmax preceded the QRS onset by 14 ms at the EAS in the unipolar‐based map, while the first rapid bipolar deflection occurred 20 ms before the QRS onset at the EAS in the bipolar‐based map. The spatial distance between the EASs on the two maps was measured at 4.5 mm.

### 3.3 VPCs Originating From the ASC

**Figure 4:**
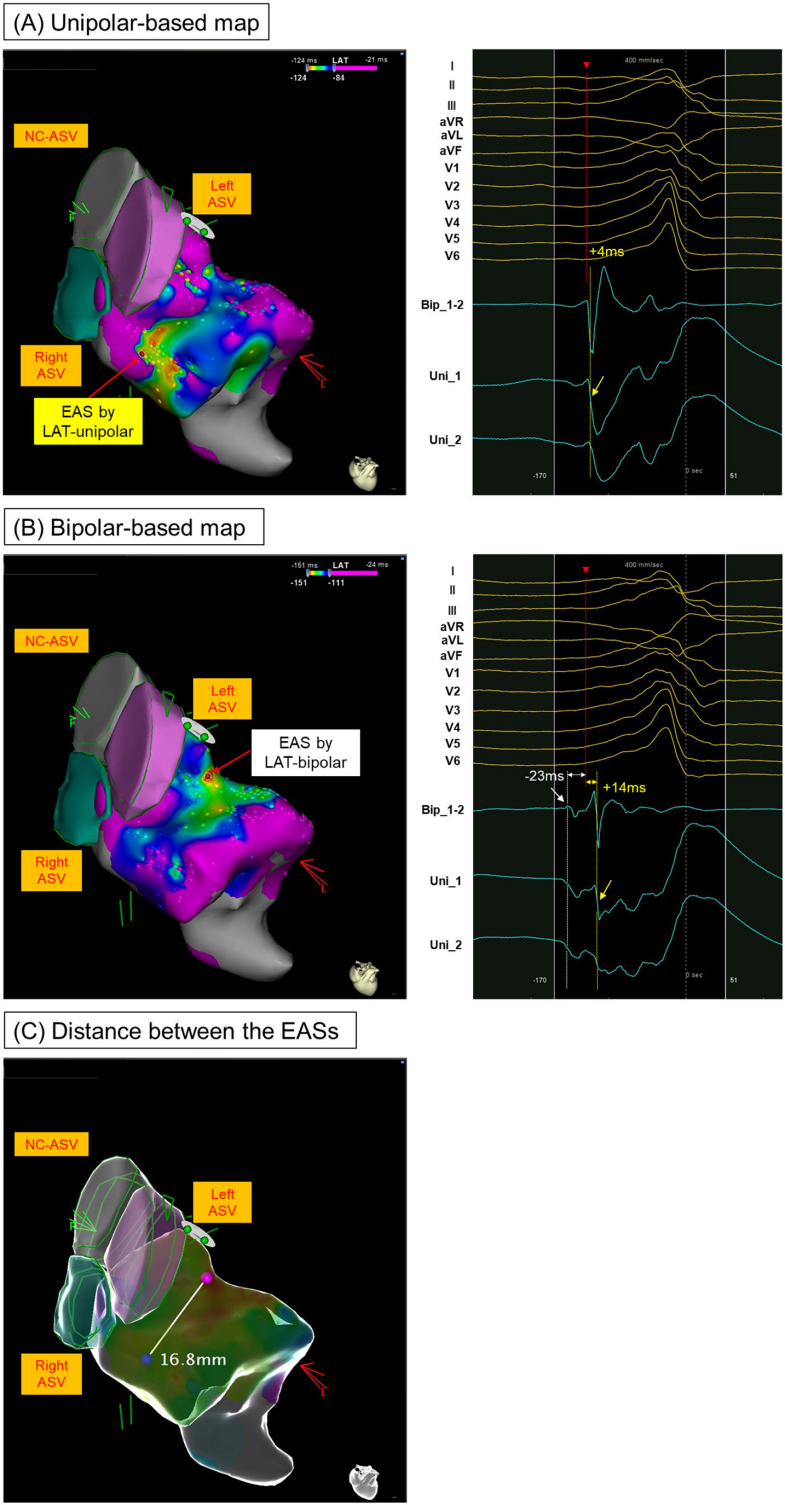
An example of the VPC case arising from ASC where ablation resulted in immediate suppression. (A) The red arrow in the left panel indicates the EAS in the unipolar‐based activation map. The right panel shows the intracardiac signals at this EAS. The unipolar −dV/dTmax (yellow dotted line) occurred 4 ms after the QRS onset. (B) The red arrow in the left panel indicates the EAS in the bipolar‐based activation map. The right panel shows the intracardiac signals at this EAS. The first bipolar deflection (white dotted line) precedes the QRS onset by 23 ms, corresponding to the first unipolar deflection. The unipolar −dV/dTmax occurs 37 ms later than this first bipolar deflection (i.e., 14 ms later than the QRS onset). (C) The blue and pink tags denote the EASs in the unipolar‐ and bipolar‐based activation maps, respectively. The distance between these points is 16.8 mm. ASV = aortic sinus of Valsalva; NC = non‐coronary; other abbreviations as in Figures 1, 2, 3.

Figure 4 shows a VPC originating from the ASC. In the unipolar‐based activation map, the unipolar −dV/dTmax at the EAS occurred 4 ms after the QRS onset. In contrast, the bipolar‐based activation map showed that the earliest bipolar potential preceded the QRS onset by 23 ms. Notably, at this point, the system automatically annotated the unipolar −dV/dTmax, which coincided with far‐field ventricular activity occurring 14 ms after the QRS onset. This resulted in significant spatial discordance, with the unipolar and bipolar EASs located 16.8 mm apart.

### 3.4 Distances Between the EAS of Unipolar‐ and Bipolar‐Based Maps

**Figure 5:**
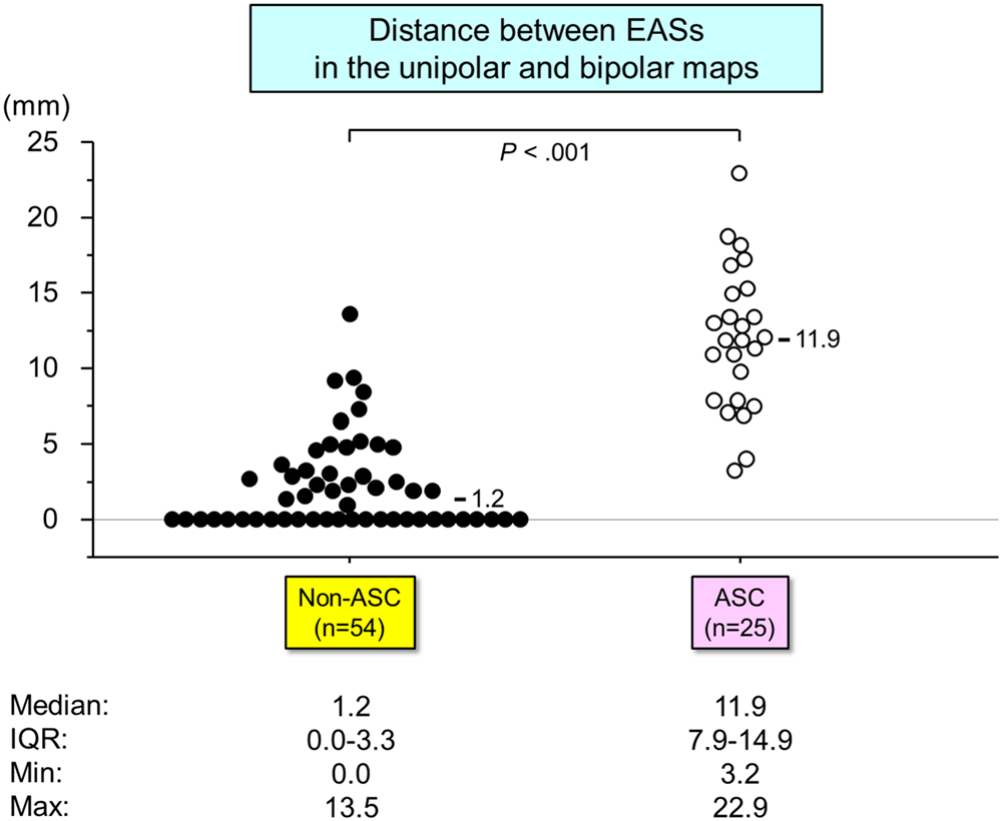
Distribution of the distance between EASs in the unipolar‐ and bipolar‐based maps between ASC versus non‐ASC origin VPCs. Abbreviations as in Figures 2 and 3. *Remained significant with a Bonferroni correction (*p* < 0.0083).

**Figure 6:**
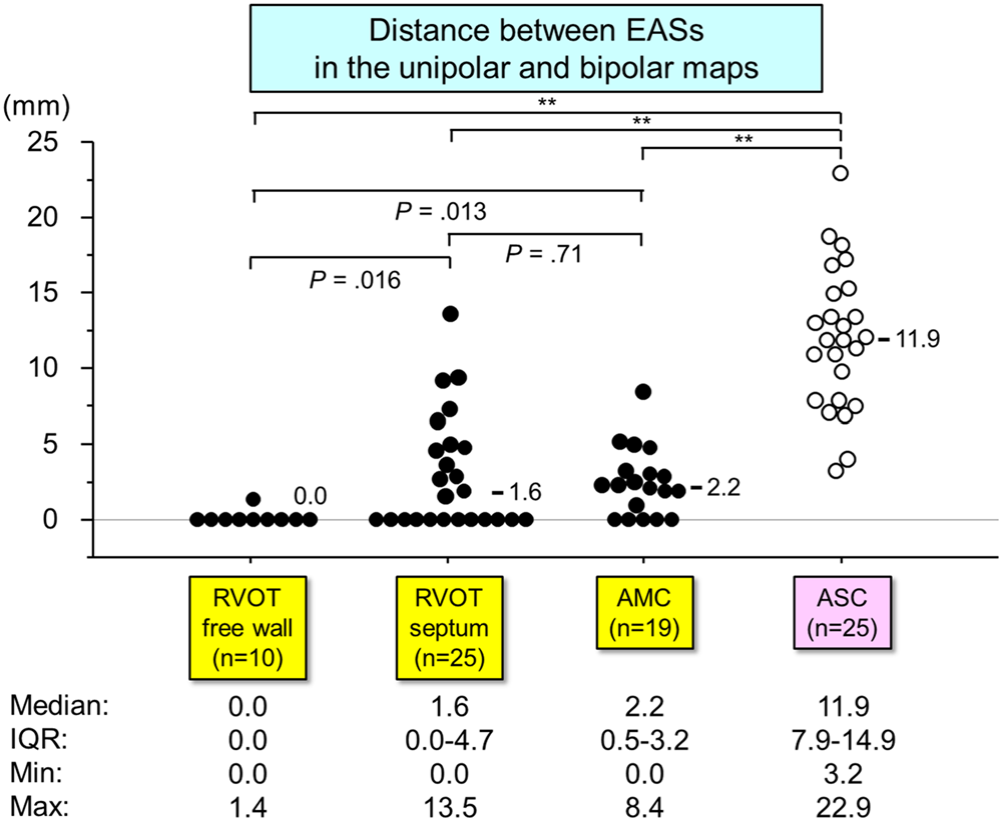
Distribution of the distance between EASs in the unipolar‐ and bipolar‐based maps depending on the successful ablation site. Abbreviations as in Figures 2 and 3. *Remained significant with a Bonferroni correction (*p* < 0.0083). **p < 0.001.

The distances between the EASs in the unipolar‐ and bipolar‐based maps were compared across two patient groups: ASC and non‐ASC (Figure 5). The distance between the EASs was significantly larger in ASC‐origin VPCs compared to non‐ASC‐origin VPCs (median: 11.9 mm [IQR: 7.9–14.9] vs. 1.2 mm [IQR: 0.0–3.3], *p* < 0.001). Seventeen of 25 (68%) VPCs from the ASC group exhibited a discordance of greater than 10 mm between the unipolar and bipolar EASs. For non‐ASC‐origin VPCs, the EAS distance was smaller in VPCs originating from the RVOT free wall (*n* = 10) (median: 0 mm [IQR: 0–0]) compared to those from the RVOT septum (median: 1.6 mm [IQR: 0–4.7], *p* = 0.016) and AMC (median: 2.2 mm [IQR: 0.5–3.2], *p* = 0.013) (Figure 6). Five of 25 (25%) VPCs from the RVOT septum and 2 of 19 (10.5%) from the AMC exhibited EAS discordances greater than 5 mm.

### 3.5 Timing of the Bipolar First Deflection and Unipolar −dV/dTmax

The temporal relationship between the bipolar and unipolar potentials at the endocardial bipolar EAS, which corresponds to the site of successful ablation, is illustrated in Table 2. The inter‐observer and intra‐observer reproducibility for the manual annotation of the timing of the first rapid bipolar deflection, which corresponds to the negative unipolar deflection, was found to be satisfactory, with an inter‐observer ICC of 0.944 and an intra‐observer ICC of 0.963.

**Table 2:** Relationship between bipolar and unipolar electrograms at the site of successful ablation.

Variable	Bipolar to QRS (ms)		Unipolar −dV/dTmax to QRS (ms)		Bipolar to unipolar −dV/dTmax (ms)	
	Values	*p* value	Values	*p* value	Values	*p* value
ASC origin VPC (*n* = 25)^a^	27.0 (22.0 to 35.0)^d^	0.038	−19.0 (−24.0 to −7.0)^b,c,d^	< 0.001	40.0 (34.0 to 53.0)^b,c,d^	< 0.001
Non‐ASC origin VPC (*n* = 54)	22.5 (19.0 to 27.0)		19.0 (‐0.75 to 35.0)		9.5 (0.0 to 20.8)	
(Non‐ASC origin subgroup)	Values		Values		Values	
RVOT free wall (*n* = 10)^b^	24.0 (20.5 to 26.8)		21.5 (20.0 to 25.3)^a,d^		0.0 (0.0 to 3.0)^a,c,d^	
RVOT septum (*n* = 25)^c^	24.0 (20.0 to 28.0)		19.0 (0.0 to 23.0)^a^		9.0 (0.0 to 21.0)^a,b^	
AMC (*n* = 19)^d^	20.0 (17.0 to 26.0)^a^		11.0 (−12.5 to 19.0)^a,b^		11.0 (10.0 to 28.5)^a,b^	

*Note:* Values are presented as median (interquartile range). A superscript of the value indicates that the comparison with its group is significantly different (*p* < 0.05). For instance, if a value has a superscript of “a” in this table, it means a significant difference from that in the group of “a” (vs. ASC origin VPC).

Abbreviations: −dV/dTmax = maximal negative derivative of the extracellular unipolar potential; AMC = aortomitral continuity; ASC = aortic sinus cusp; RVOT = right ventricular outflow tract; VPC = ventricular premature contraction.

## 4 Discussion

The present study compared the EASs of VPCs originating from the right and left ventricular OTs using two methods: unipolar −dV/dTmax and the first deflection of the bipolar electrogram. The major findings are as follows:

(1) The distance between EASs was significantly larger in ASC‐origin VPCs (median: 11.9 mm) compared to non‐ASC‐origin VPCs (median: 1.2 mm).

(2) In non‐ASC‐origin VPCs, the distance between EASs was smaller for VPCs originating from the RVOT free wall (median: 0 mm) compared to those from the RVOT septum (median: 1.6 mm) and AMC (median: 2.2 mm). Five of 25 (25%) VPCs originating from the RVOT septum and 2 of 19 (10.5%) from the AMC exhibited an EAS discordance greater than 5 mm.

(3) The discordance between EASs in the two maps (unipolar‐ and bipolar‐based) varied depending on the site of VPC origin.

Idiopathic ventricular arrhythmias commonly arise from the ventricular OT regions (RVOT and LVOT), which are transitional zones between the ventricles and the great arteries, containing specialized conduction tissue and cardiac muscle cells. The complex anatomical features of these OTs contribute to the propensity for ectopic activity [6]. The anatomical characteristics in the OT regions influence the effectiveness of radiofrequency energy in eliminating VPCs. The precise location of VPC origin is crucial for therapeutic strategies, including the power and duration of radiofrequency application.

A previous study indicated that the automated algorithm based on unipolar −dV/dTmax for LAT annotation during VPC ablation achieved similar clinical outcomes with greater procedural efficiency compared to conventional manual annotation [7]. However, recent studies have shown that unipolar −dV/dTmax is less ideal than bipolar electrograms for identifying VPC origins when they arise from intramural sources [3, 4]. In this context, we showed spatial differences in the EASs of the targeted VPC between an automated annotation algorithm based on unipolar −dV/dTmax versus a manual annotation using local bipolar potential, depending on the site of the VPC origin among the right and left OTs.

In the present study, five of 25 (25%) VPCs originating from the RVOT septum exhibited a greater than 5 mm difference between the unipolar and bipolar EASs. All five patients required prolonged radiofrequency application (over 10 s). These findings suggest that some VPCs may originate from intramural locations through anisotropic myocardial conductive fibers. Conversely, the distance between EASs was smaller (median: 0 mm [range: 0.0–1.4]) for VPCs originating from the RVOT free wall compared to those from the RVOT septum (median: 1.6 mm [range: 0.0–13.5]). The myocardium in the anterior and sub‐pulmonary valve regions of the RVOT (free wall) is relatively thin, while the posterior infundibular part (RVOT septum), which adheres to the anterior LVOT, is thicker. These anatomical features may explain why VPCs near these areas can have varying depths, potentially leading to misinterpretation of their origins as being from the aortic root rather than the RVOT itself. Regarding the temporal difference, the time from bipolar to unipolar −dV/dTmax was significantly longer in VPCs originating from the RVOT septum compared to those from the RVOT free wall (median: 9.0 ms [range: 0.0–21.0] vs. median: 0 ms [range: 0.0–3.0], *p* < 0.001), which aligns with findings from a previous study [8]. For VPCs originating from the AMC, the distance between EASs in the unipolar‐ and bipolar‐based maps was larger (median: 2.2 mm [range: 0.0–8.4]) compared to those from the RVOT free wall. The AMC is a fibrous curtain that connects the aortic and mitral valve leaflets, supported by a triangular region of myocardial tissue that is a known site for idiopathic ventricular arrhythmias [9]. It extends from the left fibrous trigone to the right fibrous trigone. This fibrous structure varies in thickness along its length, which may influence the depth of arrhythmogenic foci. Previous studies have indicated that atrioventricular junctional tissue, if it fails to regress, can become arrhythmogenic later in life [10, 11]. The distribution of these cells at varying depths could lead to different VPC origins, contributing to the differences observed between the EASs in the unipolar‐ and bipolar‐based maps, suggesting the existence of a dead‐end tract and/or anisotropic myocardial conduction.

Similarly, a recent Langendorff preparation study, combined with in silico verification, demonstrated that unipolar endocardial/epicardial EAS can become increasingly misleading as the depth of the focal source origin increases. Anisotropic transmural activation may account for the increased displacement of the endocardial EAS with greater focal source depth, as the activation spread to the surface is amplified with increased intramural depth [12].

In our study population, ASC‐origin VPCs exhibited a larger spatial discordance (median: 11.9 mm [range: 3.2–22.9]) between the unipolar and bipolar maps. In addition to the aforementioned anisotropic conduction, the presence of a preferential pathway connecting the VPC origin to the myocardial breakout site around the ASC may contribute to the greater spatial differences in EASs observed between the two mapping methods [13, 14, 15]. Myocardial extensions may also occur in the intercuspal clefts in addition to within the cusps. The spatial discordance identified in this study may be explained by anisotropic conduction along the preferential pathway, characterized by sparse and/or insulated myocardial fibers. The larger spatial discordance observed in ASC‐origin VPCs highlights the greater need for careful interpretation of activation maps that are automatically generated based on the unipolar −dV/dTmax of the local potential. This is crucial for accurately identifying the VPC origin and ensuring effective ablation [8].

## 5 Limitations

This study is a single‐center, retrospective analysis involving a limited number of patients, which presents several limitations beyond those associated with the nature of the study population. First, defining the “true” VPC focus originating from the ventricular OTs can sometimes be challenging, particularly due to the anatomical proximity of sites such as the ASC, AMC, and left ventricular summit. VPCs from these locations may have been successfully ablated by applying radiofrequency energy from multiple locations. In cases where RF ablation was performed at multiple sites, particularly involving both the RVOT and LVOT, the final successful ablation site may have been affected by prior ablation lesions. In this study, we classified the VPC origin based on the site where VPC elimination was ultimately achieved, assuming it to be the closest location to the true VPC origin. Second, we restricted our analysis to patients who underwent mapping and ablation using the CARTO system, which employs an automated annotation algorithm based on unipolar −dV/dTmax. Therefore, the results may not be generalized to other 3D mapping systems. Third, the distance between EASs was smaller in VPCs originating from the RVOT free wall compared to those from the RVOT septum (*p* = 0.016) and AMC (*p* = 0.013). However, these differences did not achieve statistical significance after applying a Bonferroni correction. Lastly, it is important to recognize that bipolar electrogram can be influenced by the direction of wavefront propagation, the size of the electrodes, and interelectrode spacing, which may affect the morphology and measurements of the electrograms [1].

## 6 Conclusion

The spatial concordance of the EASs between the unipolar‐ and bipolar‐based maps varied depending on the site of VPC origin. This variability should be taken into account when using automated annotation algorithms based on unipolar −dV/dTmax in 3D mapping systems.

## Data Availability

The data that support the findings of this study are available from the corresponding author upon reasonable request.
